# Pseudogenes, RNAs and new reproducibility norms

**DOI:** 10.7554/eLife.56397

**Published:** 2020-04-21

**Authors:** George A Calin

**Affiliations:** 1 Translational Molecular Pathology Department, University of Texas MD Anderson Cancer CenterHoustonUnited States; 2 Center for RNA Interference and Non-Coding RNAs, University of Texas MD Anderson Cancer CenterHoustonUnited States

**Keywords:** Reproducibility Project: Cancer Biology, replication, metascience, PTEN, noncoding RNA, pseudogene, Human

## Abstract

The partial success of a study to reproduce experiments that linked pseudogenes and cancer proves that understanding RNA networks is more complicated than expected.

**Related research article** Kerwin J, Khan I, Reproducibility Project: Cancer Biology. 2020. Replication Study: A coding-independent function of gene and pseudogene mRNAs regulates tumour biology. *eLife* **9**:e51019. doi: 10.7554/eLife.51019

Of all the molecules involved in the flow of genetic information within any biological system, RNA is the oldest (Crick, 1970). This means that RNA molecules can interact with other RNAs and with all the other molecules that appeared later in evolution, including DNA molecules, proteins and lipids (Fabbri et al., 2019). RNA further evolved into coding RNA molecules (which are translated into proteins) and non-coding RNA molecules (which are not translated, but can still perform a number of other roles within cells; Pang et al., 2006). The most widely studied non-coding RNAs are the short transcripts called microRNAs that can silence a molecule of messenger RNA and prevent it from being translated into a protein. Relatively little studied, on the other hand, are non-coding RNAs called pseudogenes: a pseudogene is an RNA molecule that has been transcribed from a DNA segment that resembles a protein-coding gene but, for various reasons, this DNA is never expressed as a protein.

In 2010 researchers at the Beth Israel Deaconess Medical Center, Harvard University and the Memorial Sloan Kettering Cancer Center reported some surprising results about a pseudogene that is related to PTEN, a gene that codes for a protein that can suppress tumors (Poliseno et al., 2010). Mutations in this gene are linked to a number of cancers. Poliseno et al. reported that the pseudogene, which is called PTENP1, is biologically active and can regulate the levels of PTEN in cells via the direct interaction with a number of different microRNAs.

This report changed the dogmatic view of pseudogenes as relicts of genomic evolution: rather, by retaining multiple sites that can bind microRNAs, pseudogenes can act as decoys for their functional counterpart. The results of Poliseno et al. therefore provided support for a theory in which RNAs are regulated by other RNAs (Salmena et al., 2011; please see Thomson and Dinger (2016) for a balanced description of this theory, which is called the 'competing endogenous RNA theory'). Moreover, the results in the 2010 paper showed that pseudogenes can influence genes that are involved in cancer.

In 2015, as part of the Reproducibility Project: Cancer Biology, Khan et al. published a Registered Report which explained in detail how they would seek to replicate four of these experiments (Khan et al., 2015a; somewhat unusually, this report has been corrected twice: Khan et al. (2015b); Khan et al. (2015c). The results of these experiments have now been published as a Replication Study (Kerwin et al., 2020). As with a number of other Replication Studies in this project, some of the original results have been reproduced and some have not. Moreover, as we will describe below, the Replication Study does not contain data for one of the four experiments (although these data will be made available at https://osf.io/fjdtn/). Unfortunately, this was probably the most important of the experiments. First, however, we will discuss the experiments for which data are reported.

Poliseno et al. reported that the depletion of PTEN and/or PTENP1 increased the proliferation of DU145 prostate cancer cells, compared to administration of non-targeting siRNA, by an amount that was statistically significant (Figure 2F in the 2010 paper). The Replication Study also reports a similar increase in proliferation, and while it is not statistically significant, it supports the idea that pseudogenes can have a functional role in human cancers. Moreover, a study published by researchers at the University of Michigan in 2012 confirmed that transcribed pseudogenes are an important contributor in the transcriptional landscape of cancer cells (Kalyana-Sundaram et al., 2012).

The original study reported that overexpression of PTEN 3’UTR increased PTENP1 levels in DU145 cells (Figure 4A), whereas the Replication Study reports that it does not. As the level of the 3’UTR expression was not determined in either study, it is not possible to compare the amount of 3’UTR molecules used by the two groups, so it is difficult to make meaningful comparisons. However, the original study and the Replication Study both found that overexpression of PTEN 3’UTR led to a statistically significant decrease in the proliferation of DU145 cells compared to controls.

In the original study Poliseno et al. reported that two microRNAs – miR-19b and miR-20a – suppress the transcription of both PTEN and PTENP1 in DU145 prostate cancer cells (Figure 1D), and that the depletion of PTEN or PTENP1 led to a statistically significant reduction in the corresponding pseudogene or gene (Figure 2G). Neither of these effects were seen in the Replication Study. There are many possible explanations for this. For example, although both studies used DU145 prostate cancer cells, they did not come from the same batch, so there could be significant genetic differences between them: see Andor et al. (2020) for more on cell lines acquiring mutations during cell cultures. Furthermore, one of the techniques used in both studies – quantitative real-time PCR – depends strongly on the reagents and operating procedures used in the experiments. Indeed, there are no widely accepted standard operating procedures for this technique, despite over a decade of efforts to establish such procedures (Willems et al., 2008; Schwarzenbach et al., 2015).

What are the take-home messages from this Replication Study? One is the importance of fruitful communication between the laboratory that did the initial experiments and the lab trying to repeat them. The lack of such communication – which should extend to the exchange of protocols and reagents – was the reason why the experiments involving microRNAs could not be reproduced. The original paper did not give catalogue numbers for these reagents, so the wrong microRNA reagents were used in the Replication Study. The introduction of reporting standards at many journals means that this is less likely to be an issue for more recent papers. Another take-home message is that it is finally time for the research community to make raw data obtained with quantitative real-time PCR openly available for papers that rely on such data. This would be of great benefit to any group exploring the expression of the same gene/pseudogene/non-coding RNA in the same cell line or tissue type.

The true power of the Reproducibility Project is not restricted to what it can tell us about the robustness of papers in the field of cancer biology: rather, it should make researchers in the field – and the wider scientific community – realize that we can and should establish new standards and norms to make data freely available and comparable.

## Note

George A Calin was the Reviewing Editor for the Registered Report (Khan et al., 2015a) and the Replication Study (Kerwin et al., 2020).

## References

[bib1] Andor N, Lau BT, Catalanotti C, Sathe A, Kubit M, Chen J, Blaj C, Cherry A, Bangs CD, Grimes SM, Suarez CJ, Ji HP (2020). Joint single cell DNA-seq and RNA-seq of gastric cancer cell lines reveals rules of *in vitro* evolution. NAR Genomics and Bioinformatics.

[bib2] Crick F (1970). Central dogma of molecular biology. Nature.

[bib3] Fabbri M, Girnita L, Varani G, Calin GA (2019). Decrypting noncoding RNA interactions, structures, and functional networks. Genome Research.

[bib4] Kalyana-Sundaram S, Kumar-Sinha C, Shankar S, Robinson DR, Wu YM, Cao X, Asangani IA, Kothari V, Prensner JR, Lonigro RJ, Iyer MK, Barrette T, Shanmugam A, Dhanasekaran SM, Palanisamy N, Chinnaiyan AM (2012). Expressed pseudogenes in the transcriptional landscape of human cancers. Cell.

[bib5] Kerwin J, Khan I, Reproducibility Project: Cancer Biology (2020). Replication Study: A coding-independent function of gene and pseudogene mRNAs regulates tumour biology. eLife.

[bib6] Khan I, Kerwin J, Owen K, Griner E, Reproducibility Project: Cancer Biology (2015a). Registered Report: A coding-independent function of gene and pseudogene mRNAs regulates tumour biology. eLife.

[bib7] Khan I, Kerwin J, Owen K, Griner E, Reproducibility Project: Cancer Biology (2015b). Correction: Registered Report: A coding-independent function of gene and pseudogene mRNAs regulates tumour biology. eLife.

[bib8] Khan I, Kerwin J, Owen K, Griner E, Reproducibility Project: Cancer Biology (2015c). Correction: Registered Report: A coding-independent function of gene and pseudogene mRNAs regulates tumour biology. eLife.

[bib9] Pang KC, Frith MC, Mattick JS (2006). Rapid evolution of noncoding RNAs: lack of conservation does not mean lack of function. Trends in Genetics.

[bib10] Poliseno L, Salmena L, Zhang J, Carver B, Haveman WJ, Pandolfi PP (2010). A coding-independent function of gene and pseudogene mRNAs regulates tumour biology. Nature.

[bib11] Salmena L, Poliseno L, Tay Y, Kats L, Pandolfi PP (2011). A ceRNA hypothesis: The Rosetta Stone of a hidden RNA language?. Cell.

[bib12] Schwarzenbach H, da Silva AM, Calin G, Pantel K (2015). Data normalization strategies for microRNA quantification. Clinical Chemistry.

[bib13] Thomson DW, Dinger ME (2016). Endogenous microRNA sponges: evidence and controversy. Nature Reviews Genetics.

[bib14] Willems E, Leyns L, Vandesompele J (2008). Standardization of real-time PCR gene expression data from independent biological replicates. Analytical Biochemistry.

